# Variations in carbapenem resistance associated with the VIM-1 metallo-β-lactamase across the Enterobacterales

**DOI:** 10.1099/mic.0.001646

**Published:** 2026-01-21

**Authors:** Mia Rondinelli, Sabhjeet Kaur, Owen A. Ledwell, Henry Wong, Prameet M. Sheth, George C. diCenzo

**Affiliations:** 1 Department of Biology, Queen’s University, Kingston, ON, Canada; 2 Division of Microbiology, Kingston Health Sciences Centre, Kingston, ON, Canada; 3 Department of Pathology and Molecular Medicine, Queen’s University, Kingston, ON, Canada; 4 Department of Biology, McMaster University, Hamilton, ON, Canada

**Keywords:** adaptive laboratory evolution, antimicrobial resistance, carbapenem, epistasis, VIM-1, whole-genome sequencing

## Abstract

The VIM-1 metallo-β-lactamase enzyme, encoded within class 1 integrons, is found in Gram-negative clinical isolates worldwide and has been linked to outbreaks of bacterial pathogens in nosocomial settings. Six *vim-1^+^* clinical isolates, from the genera *Escherichia*, *Klebsiella* and *Enterobacter*, were obtained from Kingston, Ontario, Canada. Whole-genome sequencing revealed that *vim-1* was plasmid-borne in all strains and situated as the first gene in In916 or In110 integrons. Analysis of related plasmids suggested that these *vim-1*-containing plasmids are globally disseminated and have spread via horizontal gene transfer and autochthonous vertical spread within Ontario. Interestingly, the MICs of ertapenem and meropenem, two clinically relevant carbapenem antibiotics, against these six isolates varied more than tenfold, suggesting that the effects of VIM-1 are dependent on the genomic content of the host microbe. Introducing *vim-1* into three common *Enterobacterales* laboratory strains was not sufficient to confer resistance to ertapenem and meropenem. Instead, adaptive laboratory evolution of the *vim-1*^+^ laboratory strains revealed that *vim-1*-mediated carbapenem resistance in these strains was dependent on epistatic interactions with *ompC* mutations, likely due to decreased outer membrane permeability to these antibiotics. Together, these results provide additional support for the role of gene epistasis in modulating the antimicrobial resistance phenotypes of acquired resistance genes, as well as previous results suggesting that the presence of a β-lactamase gene is insufficient to confer strong resistance to carbapenems without being paired with reduced outer membrane permeability.

## Data Availability

A National Center for Biotechnology Information (NCBI) BioProject (accession PRJNA1305022) was created to hold sequencing data associated with this study. The ONT and Illumina data used to generate the whole genome sequences were deposited in the Sequence Read Archive (SRA) under accessions SRR34970224 to SRR34970235. The annotated genome sequences of the six clinical isolates were deposited under BioProject accession PRJNA1305022. At the time of publication, the annotated genomes are on hold with NCBI due to the 2025 US government shutdown, and thus they have also been made available via GitHub (https://github.com/diCenzo-Lab/015_2025_VIM-1_analyses). All code to repeat the computational analyses reported in this study is available through GitHub (https://github.com/diCenzo-Lab/015_2025_VIM-1_analyses).

## Introduction

Antimicrobial-resistant pathogens are three times more likely to result in mortality than their susceptible counterparts [1], resulting in an estimated global death toll of 1.27 million in 2019 alone [2]. In bacteria, horizontal gene transfer (HGT) facilitates the exchange of antimicrobial resistance genes (ARGs) across diverse bacterial populations and the collection of these genes into conjugative transposons [3] and plasmids [4]. These, in turn, disseminate quickly across microbial ecosystems via HGT [5], occasionally recombining with each other along the way [6]. Transposons can additionally exacerbate antimicrobial resistance (AMR) by inserting into promoter regions, activating genes associated with conjugation and improving overall conjugation frequency [7].

While HGT plays a role in ARG dissemination and evolution, there appear to be limits to how far ARGs can spread, suggesting interplay between the chromosomal genome and acquired genes [8]. Studies investigating the dissemination of resistance to antimicrobial peptides (AMPs) and antibiotics have noted that the transfer of ARGs occurs at a higher rate between more closely related bacteria [9,10]. In one case, genes encoding AMPs from gut microbiota could not confer resistance when transferred to *Escherichia coli* [10]. In others, the transfer of ARGs from distantly related species (such as *Shewanella* spp.) to *E. coli* resulted in toxicity and cell death [11]. This is likely due to the dependence of resistance mechanisms on host physiology, suggesting that there are phylogenetic barriers that may limit the spread of certain ARGs [11,12].

Additionally, there are examples of compensatory mutations increasing the fitness of bacterial strains that have evolved AMR mechanisms. Knopp and Andersson [13] found that *E. coli* could overcome the fitness cost of losing outer membrane proteins C and F through compensatory mutations to pathways producing other porins. There is also evidence that epistatic relationships exist between acquired and chromosomal ARGs. For instance, Silva *et al.* [14] found that, occasionally, plasmids carrying resistance genes confer a selective advantage to strains carrying ARGs on their chromosome in the absence of antibiotics.

Surveillance data from across Southern Ontario identified the emergence of clinical isolates in Gram-negative *Enterobacterales* carrying the Verona Integron-encoded Metallo-β-lactamase (VIM) [15] that was first identified in Europe [16,19]. Unlike other metallo-β-lactamases (MBLs) identified in this Canadian surveillance programme [17], VIM-positive patients had none of the ‘classical’ risk factors seen in patients with other MBLs including documented travel history outside of Canada or hospital admission, suggesting local acquisition. The dissemination of these pathogens is likely through undetected colonization and transmission amongst patients in acute care [17] or acquisition via unknown community exposure.

Like other MBLs, but unlike extended-spectrum β-lactamases (ESBLs), VIM can hydrolyse carbapenem antibiotics, which are often considered as a last-resort antibiotic. Of all VIM genes, *vim-1* is the most common gene variant globally [20]. Like other B1 MBLs, the structure of VIM-1 displays an overall αβ/βα fold with the Zn^2+^ centre forming the active site situated in a shallow groove formed by the interface of the two αβ domains [21]. The *vim-1* gene is generally carried as part of class 1 integrons, which are major contributors to AMR through their ability to capture and express a diverse range of ARGs [22]. Class 1 integrons can be found embedded within an extremely broad range of plasmids, including plasmids of incompatibility (Inc) groups IncA [23], IncC [24], IncH12 [17], IncFII [25], IncN [26], IncP [27], IncL/M [28] and IncR [17]. There is also evidence of these integrons integrating into the host chromosome [29]. This promiscuity results in highly mobile genetic apparatuses that can spread horizontally across a broad range of bacterial species.

Here, we report the sequencing and phenotypic characterization of six *Enterobacterales* clinical isolates collected at the Kingston Health Sciences Center, all of which were positive for *vim-1*. We demonstrate that the MICs of the carbapenem antibiotics ertapenem and meropenem varied more than tenfold across the isolates, suggesting that the effects of *vim-1* are dependent on the genomic content of the host microbe. Introduction of *vim-1* into common laboratory strains resulted in little ertapenem resistance. However, adaptive laboratory evolution (ALE) led to massive increases in the level of *vim-1*-mediated ertapenem resistance, which we show was due to a combination of increased *vim-1* gene dosage and epistatic interactions with mutations of the *ompC* gene encoding an outer membrane protein.

## Methods

### Bacterial strains and growth conditions

Bacterial strains used in this work are listed in Table 1. Strains were routinely grown using lysogeny broth (LB) medium (10 g l^−1^ tryptone, 5 g l^−1^ yeast extract, 5 g l^−1^ sodium chloride) and Mueller–Hinton (MH) medium (Sigma-Aldrich; Product No. 90922). Gentamicin (10 µg ml^−1^ in liquid media, 20 µg ml^−1^ in solid media) was used to maintain pBBR1mcs-5 plasmids in transformed strains. Super optimal broth with catabolite repression (SOC) (20 g l^−1^ tryptone, 5 g l^−1^ yeast extract, 10 mM NaCl, 2.5 mM KCl, 10 mM MgSO4, 10 mM CaCl2, 20 mM glucose) was used as a recovery medium following transformation by electroporation. All strains were grown at 37 °C. When required, 5-bromo-4-chloro-3-indolyl-β-d-galactopyranoside (X-gal) was added to the media at a final concentration of 40 µg ml^−1^.

**Table 1.** Bacterial strains and plasmids

Strain/plasmid	Species	Characteristics	Year isolated	Source	Reference
DH5α	*E. coli*	F^–^*endA1 glnV44 thi-1 recA1 relA1 gyrA96 deoR nupG purB20* φ80d*lacZ*ΔM15 Δ(*lacZYA-argF*)U169, *hsdR17*(*r_K_*^–^*m_K_*^+^), λ^–^	na	na	Grant *et al*. [87]
MG1655	*E. coli*	F^–^ λ^–^ *ilvG*^–^ *rfb-50 rph-1*	na	na	Guyer *et al*. [88]
F5446	*E. coli*	Clinical isolate from KHSC	2015	Blood and urine	This study
S2568	*E. coli*	Clinical isolate from KHSC	2019	Rectal screen and urine	This study
F48994	*Enterobacter hormaechei*	Clinical isolate from KHSC	2018	Urine	This study
H17629	*E. hormaechei*	Clinical isolate from KHSC	2020	Wound swab	This study
H70375	*E. hormaechei*	Clinical isolate from KHSC	2015	Blood culture	This study
T64870	*Klebsiella pneumoniae*	Clinical isolate from KHSC	2017	Sputum culture	This study
KP5022	*Klebsiella grimontii*	*hisD2 hsdRI nif* ^+^	na	na	Liu *et al*. [89]
pBBRmcs-5	–	Broad host range vector; pLac promoter; RK2 mobilizable; Gm^R^	na	na	Kovach *et al*. [58]

### Whole-genome sequencing, assembly, and annotation

Genomic DNA (gDNA) samples were isolated from clinical isolates using phenol-chloroform extraction [30]. Purified gDNA samples were then sequenced using an Oxford Nanopore Technologies (ONT) MinION Mk1B nanopore sequencer and the Rapid Barcoding Kit (RBK004) according to the manufacturer’s instructions. Base calling and demultiplexing were performed using GPU-enabled Guppy version 5.011+2b6dbffa5 and the High-Accuracy model (ONT). Purified gDNA samples were also sequenced at the Microbial Genome Sequencing Center (Pittsburgh, PA, USA) using an Illumina NextSeq 550 instrument with 150 bp paired-end reads.

 Genome assemblies were generated using a pipeline previously described by Duan *et al.* [31]. Programs and versions used are as follows: Flye version 2.8.3 [32], NUCmer version 4.0.0rc1 [33], Racon version 1.4.22 [34], Minimap2 version 2.20-r1061 [35], Medaka version 1.4.1 (ONT), Pilon version 1.24 [36], bwa version 0.7.17.r1198-dirty [37] and Circlator version 1.5.5 [38]. Assembly quality was checked using CheckM version 1.2.2 [39].

 Genome assemblies were annotated using the NCBI Prokaryotic Genome Annotation Pipeline build 5429 [40]. Plasmid replicons were identified in the genome assemblies using PlasmidFinder version 2.1.1 [41]. Integron-associated features were identified within the genome assemblies using IntegronFinder version 2.0.2 [42]. Resistance genes/proteins were identified in nucleotide or amino acid sequences using the Comprehensive Antibiotic Resistance Database 3.2.2 Resistance Gene Identifier 5.2.1 [43]. Genome assemblies were visualized in Mauve snapshot 2015-02-25 build 0 following alignment with progressiveMauve [44]. Taxonomic classification of strains was performed using TYGS [45].

### Identification of related *vim-1*-containing plasmids

To identify *vim-1*-containing plasmids related to those of our clinical isolates, we first downloaded the plasmid database (PLSDB) version 2021_06_23_v2 [46]. Next, each of the *vim-1*-containing plasmids of our clinical isolates was individually used as queries with BLASTn from blast version 2.10.1+ [47] to search PLSDB, and the top 20 hits for each plasmid were recorded. The exception was the *vim-1*-containing plasmid of S2568, as this plasmid was not circularized. The dereplicated top hits from each plasmid were collected from PLSDB and annotated using Prokka version 1.14.6 [48], as were the *vim-1*-containing plasmids of our isolates. Roary version 1.7.8 [49] was then used to identify shared genes between all annotated plasmids. The resulting gene presence/absence matrix was used to produce a distance matrix based on Jaccard distances using the philentropy version 0.8.0 [50] package in R version 4.3.0 [51]; this distance matrix reflects how similar each plasmid is to each other based on presence/absence of orthologous genes. The distance matrix was then used to construct a neighbour-joining dendrogram using the bionj function of ape version 5.8 [52]. Subsequently, 1,000 bootstrap replicates were constructed by subsampling the gene presence–absence matrix with replacement, and then calculating a distance matrix and a neighbour-joining dendrogram as described above. Bootstrap values were then calculated with the plotBS function of phangorn version 2.12.1 [53]. The dendrogram was visualized using iTOL [54].

### Sequence comparison of OmpC and OmpD porins

All proteins annotated as OmpC or OmpD in the genomes of the six clinical isolates, as well as *E. coli* EcGQ0079 (DH5α with a pBBR1mcs-5::*vim-1* derivative), were extracted from the proteomes. All porins were aligned using the Clustal Omega [55] EMBL-EBI webserver (ebi.ac.uk/jdispatcher/msa/clustalo) to generate a multisequence alignment and a percent identity matrix. The untrimmed alignment was used to create a phylogeny using IQ-TREE version 2.2.2.4 [56] with the Q.pfam+F+G4 model, as it was identified as the best-scoring model by ModelFinder based on Bayesian information criterion and with model search limited to the LG, WAG, JTT, Q.pfam, JTTDCMut, DCMut, VT, PMB, BLOSUM62 and Dayhoff models. Branch supports were assessed using the Shimodaira–Hasegawa-like approximate likelihood ratio test [57] and an ultrafast bootstrap analysis, with both metrics calculated from 1,000 replicates. The phylogeny and percent identity matrix were then visualized with the iTOL web server [54].

### Cloning of *vim-1*

PCR primers MR001 and MR003 (see Table S1 for all primer sequences, available in the online Supplementary Material) were used to amplify *vim-1* and the preceding *attC* site from *E. coli* F5446 using Q5 DNA polymerase [New England Biolabs (NEB)], which was then purified using a Monarch PCR and DNA Cleanup Kit (NEB). The PCR product and the pBBR1mcs-5 cloning vector [58] were individually digested with both BamHI-HF (NEB) and HindIII-HF (NEB) overnight at 37 °C; pBBR1mcs-5 was subsequently dephosphorylated using Quick CIP (NEB). Lastly, the *vim-1*-containing DNA fragments were ligated into pBBR1mcs-5 using T4 DNA ligase (NEB). Ligation products were transformed into chemically competent *E. coli* DH5α and plated on LB with gentamicin and X-gal, and correctly assembled plasmids were preliminarily identified based on blue-white screening. Plasmid DNA was purified from transformants using a Monarch Plasmid DNA Miniprep Kit (NEB), and the *vim-1* sequences were then verified using Sanger sequencing with primers M13-F and M13-R (CHU de Québec-Université Laval Research Center). Plasmids containing *vim-1* were also transformed into electrocompetent cultures of *K. grimontii* KP5022 [59] and *E. coli* MG1655 [60] via electroporation and plated on LB with gentamicin to select for the presence of the plasmid.

### Adaptive laboratory evolution

Strains of interest were grown overnight in MH broth and diluted to an OD at 600 nm (OD_600_) of 0.05 in 200 µl MH broth in 96-well microplates. Each microplate contained an ertapenem gradient, where the ertapenem concentration of adjacent wells differed by a factor of two. Microplates were taped closed, inserted into a Synergy H1 plate reader and incubated for 24 h at 37 °C with shaking. OD_600_ measurements were recorded every 15 min. Following 24 h of incubation, 2 µl from the well with the highest concentration of ertapenem that allowed growth was sub-cultured into each well of an ertapenem concentration gradient in a fresh 96-well plate. This process was repeated daily for 5–6 days (representing five to six passages). For each passage, the ertapenem concentration gradient was adjusted such that the midpoint concentration was equal to the highest concentration of ertapenem that allowed growth in the previous passage.

To test if the plasmids in the derived isolates were sufficient to produce an ertapenem resistance phenotype, plasmid DNA from the derived isolates was purified with a Monarch Plasmid DNA Miniprep Kit (NEB) and transformed into electrocompetent * E. coli* DH5α cultures via electroporation, then plated on LB with Gm to select for plasmid uptake.

 Plasmids from the strains recovered at the end of the ALE were purified using a Monarch Plasmid Miniprep Kit (NEB), and full plasmid sequences were determined using ONT sequencing by Plasmidsaurus (Louisville, KY, USA). Sequencing results were visualized using the online software Benchling (benchling.com), with alignment performed using MAFFT [61], while dot plots were generated with the online YASS tool [62]. For one ALE experiment, gDNA from an ancestral strain (EcGQ0079) and two derived lineages (EcGQ0088 and EcGQ0089) was purified and sequenced at the Microbial Genome Sequencing Center using an Illumina NextSeq 550 instrument with 150 bp paired-end reads. Illumina reads were then trimmed using BBduk version 38.96 [63] and Trimmomatic version 0.39 [64]. A reference genome was assembled using the Illumina reads of the ancestral strain and Unicycler version 0.5.0 [65] with SPAdes version 3.15.4 [66] and then annotated using Prokka version 1.14.6 [48]. Next, polymorphisms between the derived isolates and the reference genome were identified using Snippy version 4.6.0 [67] with bwa version 0.7.17-r1198-dirty [37]. Larger deletions were identified using the samtools version 1.15 coverage and depth functions [68] and the BAM files returned by Snippy.

### Antibiotic susceptibility

MICs of ceftazidime, meropenem and ertapenem were determined with ThermoFisher Oxoid M.I.C.Evaluator (M.I.C.E.) strips according to the manufacturer’s standard protocol on MH agar plates at 37 °C. Antibiotic susceptibility was determined in accordance with CLSI guidelines [69].

### Reverse transcriptase quantitative PCR

Three biological replicates of each strain of interest were inoculated into 2 ml of MH broth with relevant antibiotics (10 µg ml^−1^ of gentamicin was used for strains containing the pBBR1mcs-5 construct to stabilize the plasmid, and a sub-inhibitory concentration of 0.5 µg ml^−1^ of ertapenem was used for clinical isolates to induce *vim-1* expression) and grown overnight at 37 °C. The following day, cells were pelleted and washed with fresh media, then sub-cultured to a starting density of OD_600_=0.05, grown to a final density of OD_600_=0.5 at 37 °C, pelleted, flash frozen and stored at −80 °C. RNA was purified from cell pellets with ZymoBIOMICS RNA Miniprep Kit (Zymo Research) according to the manufacturer’s instructions, which was then treated with a TURBO DNA-*free* Kit (Thermo Fisher) following the manufacturer’s instructions, to ensure that the samples were free of contaminating DNA. Next, cDNA was synthesized from 2 µg of template RNA using the SuperScript IV VILO Master Mix Kit (Invitrogen), according to the manufacturer’s instructions.

 To examine the expression of *vim-1*, qPCR was performed using a BioRad CFX Opus 96 Real-Time PCR System. Expression of *vim-1* was normalized based on expression of the 16S rRNA gene. Standard curves were created for the 16S rRNA gene (primers: SK003 and SK004) and *vim-1* (primers: GD018 and GD019) in 16 µl reactions that included 8 µl of SsoAdvanced Universal SYBR Green qPCR Supermix (BioRad), 187.5 nM of each primer and between 0.002 and 200 ng of cDNA. The standard curves for both primer sets gave efficiencies >94% and R^2^ values>0.99 (Fig. S1). All subsequent qPCRs used 2 ng of cDNA.

## Results

### Whole-genome sequencing of six *vim-1*-positive *Enterobacterales* clinical isolates

Six multidrug-resistant clinical isolates of the order *Enterobacterales* were isolated from patients at the Kingston Health Sciences Centre, Kingston, Ontario, Canada, between 2015 and 2020. Despite all six isolates testing positive for the presence of *vim-1* based on a PCR screen, they displayed a wide range of resistance to the carbapenem antibiotics ertapenem and meropenem, with some isolates phenotypically testing as susceptible despite the presence of *vim-1* (Table 2). In contrast, all strains were equally and highly resistant to the cephalosporin antibiotic ceftazidime (Table 2).

**Table 2.** MICs of ceftazidime, meropenem and ertapenem against six clinical isolates, together with the copy numbers of *vim-1*-containing plasmids and *vim-1* expression levels

Isolate	MICs (µg ml^−1^)*			*vim-1* plasmid copy no.†	*vim-1* expression (∆Cq)‡
	Ceftazidime	Meropenem	Ertapenem		
*E. coli* F5446	≥256 (R)	6 (R)	2 (R)	1	11.8±1.0
*E. coli* S2568	≥256 (R)	0.75 (S)	3 (R)	15	13.4±0.6
*E. hormaechei* F48994	≥256 (R)	4 (R)	2 (R)	2	10.5±0.1
*E. hormaechei* H17629	≥256 (R)	0.38 (S)	0.25 (S)	1	10.4±0.4
*E. hormaechei* H70375	≥256 (R)	0.5 (S)	0.38 (S)	5	9.6±0.7
*K. pneumoniae* T64870	≥256 (R)	1 (I)	0.25 (S)	4	11.1±0.4

*MICs are marked susceptible (S), intermediate (I) or resistant (R) according to the CLSI guidelines.

†Copy number of the *vim-1*-containing plasmids are based on the copy numbers reported by the Flye assembler.

‡Values represent the average ∆Cq of three biological replicates±sd; lower numbers represent higher expression. Expression of *vim-1* was normalized based on expression of the 16S rRNA genes.

 Whole-genome sequencing, assembly and annotation were performed for all six clinical isolates. High-quality genomes were assembled for all six strains (Table S2), and taxonomic classification of the isolates indicated that F48994, H17629, and H70375 belonged to the species *E. hormaechei*, F5446 and S2568 belonged to the species *E. coli* and T64870 belonged to the species * K. pneumoniae*.

 Each of the isolates contained at least six perfect hits (100% nucleotide sequence identity) to different ARGs (Table S3). As expected, all six isolates carried the *vim-1* gene, but they also contained between 0 and 6 additional β-lactamase genes of the types ACT-24, CTX-M-15, LAP-2, OXA-1, OXA-9, TEM-1, SHV-12, and SHV-28 (Table S3). The β-lactamases ACT-24, CTX-M-15, LAP-2, and TEM-1 are not known to interact with carbapenems and therefore should not contribute to ertapenem or meropenem resistance [43]. Likewise, while some OXA-family β-lactamases are considered ESBLs, OXA-1 and OXA-9 are not known to interact with carbapenems [70], and SHV-12 (found in isolates F5446 and S2568) and SHV-28 (found in isolate T64870) are only capable of mediating carbapenem resistance in combination with major outer membrane porin modifications [71]. Although these additional β-lactamase genes likely contributed to high ceftazidime resistance in the clinical isolates, these results suggest that *vim-1* is primarily responsible for the observed carbapenem resistance.

### Genomic context of the *vim-1* genes of the six clinical isolates

**Fig. 1.**
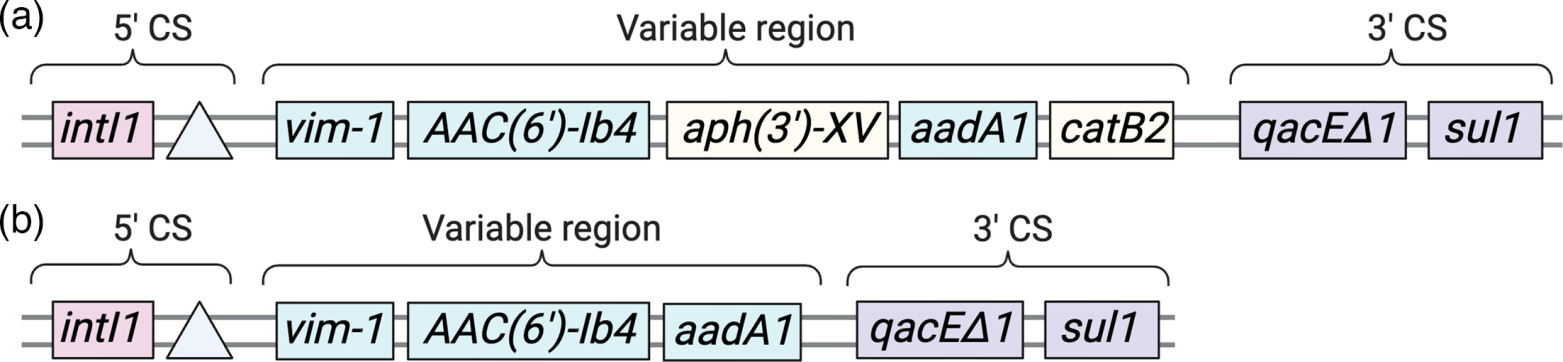
The structure of the *vim-1*-containing class 1 integrons identified in this study. (**a**) *E. coli* F5446 and *E. coli* S2568 encode VIM-1 within In916 integrons, whereas (**b**) *E. hormaechei* F48994, *E. hormaechei* H17629, *E. hormaechei* H70375 and *K. pneumoniae* T64870 encode VIM-1 within In110 integrons. (**a, b**) The 5′ conserved sequences (5′ CS), variable regions and 3′ conserved sequences (3′ CS) are indicated above each diagram. The position of *attL1* is represented as a light blue triangle, while the two genes present in In916 but absent from In110 are shown in light yellow. The diagrams are not drawn to scale.

As expected, *vim-1* was situated within an integron in each of the six clinical isolates. However, the type of integron on which *vim-1* was housed differed along species lines; the *E. coli* isolates contain In916 integrons, while the other isolates contain In110 integrons [20]. In916 and In110 are clinical class 1 integrons (Fig. 1) that both contain a 5′ conserved sequence that includes the *intI1* integrase, a 3′ conserved sequence that includes the ARGs *qacE∆1* and *sul1* and a variable region encoding additional ARGs that differs in content between In916 and In110 [43]. Notably, in all six of our strains, *vim-1* is the first gene within the variable region.

 All six *vim-*1-containing integrons were housed on plasmids rather than the bacterial chromosome. *E. coli* clinical isolates F5446 and S2568 contained *vim-1* in the context of IncA plasmids, while other isolates contained their *vim-1* in the context of IncN (*E. hormaechei* H17629), IncR (*E. hormaechei* H70375) or multi-locus IncN-IncR plasmids (*E. hormaechei* F48994, * K. pneumoniae* T64870). The copy number (as determined by the genome assembler Flye) of *vim-1-*containing plasmids varied across clinical isolates, but these variations were not correlated with *vim-1* expression expressed as ∆Cq [Spearman correlation coefficient (*rho*)=0.17; *P*=0.74] or tolerance to meropenem (*r*=−0.23; *P*=0.66) or ertapenem (*r*=0.36; *P*=0.49) (Table 2, Fig. S2). Likewise, *vim-1* expression, as determined by reverse transcriptase quantitative PCR (RT-qPCR) and expressed as ∆Cq, was not significantly correlated with relative meropenem resistance (*r*=0.54; *P*=0.30) or ertapenem resistance (*r*=0.62; *P*=0.19) resistance (Table 2, Fig. S2).

### Relationships between the *vim-*1-containing plasmids of the six clinical isolates

**Fig. 2.**
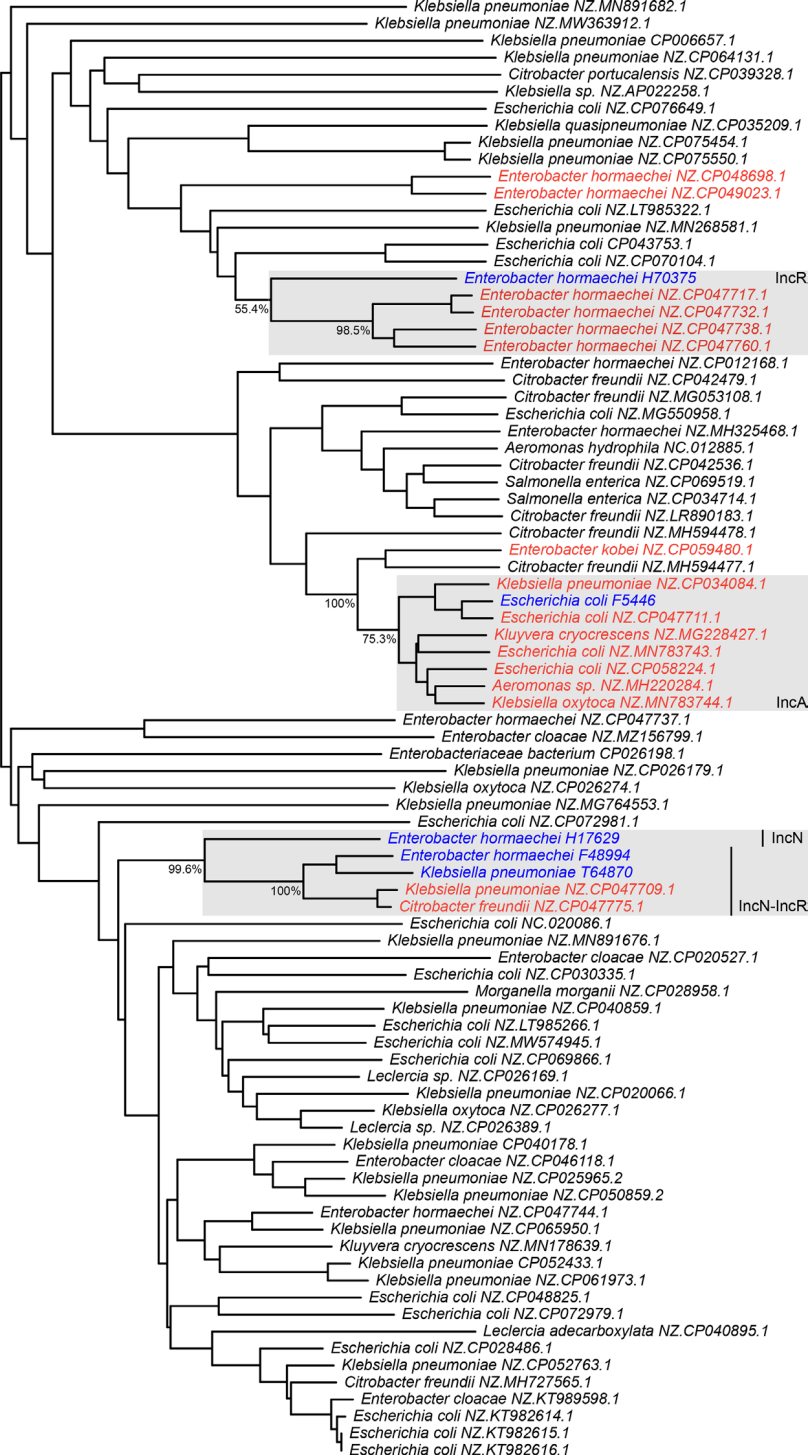
Relationships between the vim-1-containing plasmids of this study and related plasmids of the PLSDB. Plasmids related to the *vim-1*-containing plasmids identified in the six focal clinical isolates of this study were identified in PLSDB using blast, and a dendrogram was constructed from a distance matrix built on gene presence/absence data. Shown in blue are the *vim-1*-containing plasmids identified in the current study, while red is used to represent other *vim-1*-containing plasmids. Clades of interest discussed in the manuscript text are highlighted with light grey boxes, with each box labelled with the incompatibility group classification of all members of the clade; the exception is the plasmid from strain H17629, which has a different classification from the rest of the group, and thus its classification is indicated separately. Bootstrap support values based on 1,000 replicates are provided at the base of clades of interest. Plasmids are named according to the species in which the plasmid was identified, followed by the GenBank accession of the plasmid.

To explore the introduction and dissemination of *vim-1* genes within Kingston (Ontario, Canada) and the surrounding region, a mid-point-rooted, bifurcating dendrogram based on shared gene content was produced for the *vim-1*-containing plasmids of our 6 clinical isolates together with a set of 75 related plasmids (Fig. 2). Of the 81 external plasmids that were included in the analysis, 16 carried *vim-1*. Overall, the *vim-1* plasmids from our clinical isolates formed three distinct clades that also included *vim-1*-containing plasmids from isolates collected in Toronto (Ontario, Canada), suggesting three separate introductions of *vim-1* genes into the Kingston region and the spread of these genes/pathogens between Toronto and Kingston.

The *vim-1*-containing plasmids of *E. hormaechei* F48994, *E. hormaechei* H17629 and *K. pneumoniae* T64870 (which all contain *vim-1* within an In110 integron) formed a clade with two other *vim-1*-containing plasmids: pKpn13-2 (CP047709) and pCfr13-2 (CP047775). These plasmids were isolated from rectal samples of *K. pneumoniae* and *Citrobacter freundii*, respectively, by the Toronto Invasive Bacterial Diseases Network (TIBDN) from a single patient in Toronto, ON [17]. These results suggest that this *vim-1*-containing plasmid has spread between pathogens through horizontal transfer within southern Ontario. Notably, pKpn13-2 and pCfr13-2 are nested within the F48994, H17629 and T64870 cluster, suggesting that the detection of this plasmid in Toronto may have been due to spread from Kingston.

The *E. hormaechei* H70375 plasmid (which contains *vim-1* within an In110 integron) clustered with four *vim-1-*containing plasmids, albeit with only moderate bootstrap support of ~55%: pEclE2-2 (CP047717), pEcl6-3 (CP047732), pEcl5-2 (CP047738) and pEcl2-3 (CP047760). These four plasmids all came from *E. hormaechei* isolates, one of which (pEclE2-2) was an environmental isolate collected from Toronto, ON, sewage water in 2015, while the others are clinical isolates collected from hospitals by the TIBDN in Toronto, ON, between 2011 and 2014 [17]. In addition, those four plasmids all have IncR replicons and are thought to share a common ancestral IncR plasmid [17]. The plasmid from H70375, being the deepest branching lineage within this cluster, suggests that the detection of these *vim-1*-containing plasmids in Toronto may have been the result of its spread from Kingston.

Finally, there is a large cluster of *vim-1*-containing plasmids that includes the *vim-1*-containing plasmids of *E. coli* F5446 and S2568 (which contain *vim-1* within an In916 integron); the plasmid of S2568 is not shown in the dendrogram due to it being nearly identical to that of F5446 but not fully assembled. The most closely related plasmid to that from *E. coli* F5446 is pEco15-1 (CP047711), which was isolated from *E. coli* from a rectal swab gathered by the TIBDN in 2015 [17]. Interestingly, the other plasmids within this cluster are not local to Ontario and come from isolates across the globe and from multiple species. These include pR210-2 (CP034084) from a *K. pneumoniae* isolate collected at the Hong Kong Polytechnic University (unpublished), pRIVM0001_VIM-1_171012_B12 (MH220284) from a Dutch *Aeromonas* sp. sample (unpublished), p550_IncA_VIM_1 (CP058224) from an Italian *E. coli* sample [72], pGA_VIM (MN783743) from an Italian *E. coli* sample [16], pKC-BO-N1-VIM (MG228427) from an Italian *Kluyvera cryocrescens* sample [24] and pFDL_VIM (MN783744) from an Italian *Klebsiella oxytoca* sample [16].

### Expression of *vim-1* alone facilitates limited resistance to ertapenem in laboratory strains

To explore the contribution of *vim-1* to the carbapenem antibiotic ertapenem, *vim-1* of *E. coli* F5446 was PCR-amplified and expressed under the control of the pLac promoter on the plasmid pBBR1mcs-5 in three non-pathogenic lab strains: *E. coli* DH5α, *E. coli* MG1655 and *K. grimontii* KP5022. Antibiotic susceptibility tests of *E. coli* DH5α, *E. coli* MG1655 and *K. grimontii* KP5022 strains carrying an empty pBBR1mcs-5 vector confirmed that the vector itself did not contribute to ertapenem, meropenem or ceftazidime resistance (Table 3). On the other hand, the introduction of pBBR1mcs-5::*vim-1* resulted in an ~20-fold to 64-fold increase in resistance to the cephalosporin antibiotic ceftazidime (Table 3), confirming that *vim-1* was expressed and functional in all strains. Introduction of pBBR1mcs-5::*vim-1* into *K. grimontii* KP5022 also resulted in an ~40-fold increase in resistance to the carbapenem meropenem but had little impact on resistance to the carbapenem ertapenem (Table 3). Similarly, the introduction of *vim-1* into the two *E. coli* strains resulted in at most a threefold increase in resistance to meropenem and ertapenem (Table 3). In all cases, the introduction of *vim-1* did not result in any of the three strains being classified as resistant to any of the three antibiotics according to the CLSI guidelines.

**Table 3.** MICs of ceftazidime, meropenem and ertapenem against laboratory strains expressing *vim-1*, both before and after repeated passaging in growth media containing ertapenem

Species	Strain	Description	MICs (µg ml^−1^)*		
			Ceftazidime	Meropenem	Ertapenem
*E. coli*	DH5α	Wild type	0.094 (S)	0.016 (S)	0.006 (S)
*E. coli*	J243	DH5α carrying pBBR1mcs-5	0.094 (S)	0.016 (S)	0.004 (S)
*E. coli*	EcGQ0033	DH5α carrying pBBR1mcs-5::*vim-1*	2 (S)	0.032 (S)	0.006 (S)
*E. coli*	MG1655	Wild type	0.19 (S)	0.023 (S)	0.012 (S)
*E. coli*	EcGQ0110	MG1655 carrying pBBR1mcs-5	0.19 (S)	0.032 (S)	0.012 (S)
*E. coli*	EcGQ0036	MG1655 carrying pBBR1mcs-5::*vim-1*	8 (I)	0.094 (S)	0.023 (S)
*K. grimontii*	KP5022	Wild type	0.125 (S)	0.023 (S)	0.012 (S)
*K. grimontii*	KpGQ0020	KP5022 carrying pBBR1mcs-5	0.125 (S)	0.032 (S)	0.006 (S)
*K. grimontii*	KpGQ0001	KP5022 carrying pBBR1mcs-5::*vim-1*	8 (I)	0.94 (S)	0.012 (S)
*K. grimontii*	KpGQ0008	KpGQ0001 ALE-derived isolate	>256 (R)	1.5 (I)	0.004 (S)
*K. grimontii*	KpGQ0016	DH5α with pBBR1mcs-5::*vim-1* of KpGQ0008	>256 (R)	1.0 (S)	0.006 (S)
*K. grimontii*	KpGQ0009	KpGQ0001 ALE-derived isolate	>256 (R)	1.5 (I)	0.006 (S)
*K. grimontii*	KpGQ0017	DH5α with pBBR1mcs-5::*vim-1* of KpGQ0009	>256 (R)	1.0 (S)	0.006 (S)
*E. coli*	EcGQ0082	EcGQ0033 ALE-derived isolate	>256 (R)	0.25 (S)	0.006 (S)
*E. coli*	EcGQ0141	DH5α with pBBR1mcs-5::*vim-1* of EcGQ0082	>256 (R)	0.25 (S)	0.064 (S)
*E. coli*	EcGQ0137	EcGQ0082 cured of pBBR1mcs-5::*vim-1*	0.5 (S)	0.047 (S)	0.004 (S)
*E. coli*	EcGQ0083	EcGQ0033 ALE-derived isolate	>256 (R)	0.23 (S)	0.032 (S)
*E. coli*	EcGQ0142	DH5α with pBBR1mcs-5::*vim-1* of EcGQ0083	>256 (R)	0.25 (S)	0.047 (S)
*E. coli*	EcGQ0138	EcGQ0083 cured of pBBR1mcs-5::*vim-1*	1 (S)	0.047 (S)	0.012 (S)
*E. coli*	EcGQ0079	DH5α with pBBR1mcs-5::*vim-1* of KpGQ0009	>256 (R)	0.094 (S)	0.016 (S)
*E. coli*	EcGQ0088	EcGQ0079 ALE-derived isolate	>256 (R)	>32 (R)	>32 (R)
*E. coli*	EcGQ0089	EcGQ0079 ALE-derived isolate	>256 (R)	>32 (R)	>32 (R)
*E. coli*	EcGQ0143	DH5α with pBBR1mcs-5::*vim-1* of EcGQ0088	>256 (R)	0.19 (S)	0.064 (S)
*E. coli*	EcGQ0144	DH5α with pBBR1mcs-5::*vim-1* of EcGQ0089	>256 (R)	0.25 (S)	0.064 (S)
*E. coli*	EcGQ0139	EcGQ0088 cured of pBBR1mcs-5::*vim-1*	0.25 (S)	0.094 (S)	0.25 (S)
*E. coli*	EcGQ0140	EcGQ0089 cured of pBBR1mcs-5::*vim-1*	nd	nd	nd

nd, not determined due to poor growth of the strain.

*MICs are marked susceptible (S), intermediate (I) or resistant (R) according to the CLSI guidelines.

 To increase the level of resistance to ertapenem, ALE experiments were performed with *K. grimontii* KP5022 (pBBR1mcs-5::*vim-1*) and *E. coli* DH5α (pBBR1mcs-5::*vim-1*). Replicates of each strain were sub-cultured five times into increasingly higher concentrations of ertapenem, following which individual isolates were isolated from two of the replicates of each species. Antibiotic susceptibility testing indicated that all derived lineages had extremely high resistance to ceftazidime but remained sensitive to meropenem or ertapenem under the tested plate-based conditions (Table 3) despite *vim-1* expression being between 3.7- and 6.1-fold higher than in the ancestral strains (Table 4).

**Table 4.** Expression of *vim-1* in laboratory strains expressing *vim-1*, both before and after repeated passaging in growth media containing ertapenem

Species	Strain	Description	*vim-1* expression (-∆∆Cq)*
*K. grimontii*	KpGQ0001	KP5022 carrying pBBR1mcs-5::*vim-1*	0.0±1.0
*K. grimontii*	KpGQ0008	KpGQ0001 ALE-derived isolate	2.4±0.2
*K. grimontii*	KpGQ0009	KpGQ0001 ALE-derived isolate	2.6±0.4
*E. coli*	EcGQ0033	DH5α carrying pBBR1mcs-5::*vim-1*	0.0±0.3
*E. coli*	EcGQ0082	EcGQ0033 ALE-derived isolate	1.9±0.5
*E. coli*	EcGQ0083	EcGQ0033 ALE-derived isolate	2.3±0.3

*Values represent the average ∆∆Cq of three biological replicates±sd. Expression of *vim-1* was normalized based on expression of the 16S rRNA genes and then compared to the average ∆Cq of KpGQ0001 (for the *K. grimontii* strains) or EcGQ0036 (for the *E. coli* strains). The -∆∆Cq values represent the log(2) fold change compared to the reference. The difference in expression of *vim-1* in each ALE was statistically significant based on t-tests (*P*-value<0.05).

 Transfer of the pBBR1mcs-5::*vim-1* plasmids from the *K. grimontii* or *E. coli* ALE-derived isolates to otherwise WT *K. grimontii* KP5022 or *E. coli* DH5α, respectively, demonstrated that the mutation(s) responsible for the elevated ceftazidime resistance in the derived isolates were linked to the plasmids (Table 3). To identify the responsible mutation(s), we used ONT sequencing to determine the complete sequence of the pBBR1mcs-5::*vim-1* plasmids from the derived isolates and compared them to the sequence of the original pBBR1mcs-5::*vim-1* plasmid. Unexpectedly, we observed that the plasmids isolated from the derived lineages were all approximately twice the length of the original pBBR1mcs-5::*vim-1* construct, with all genes (including *vim-1*) duplicated (Fig. S3), likely because of homologous recombination. This result suggests that the increased ceftazidime resistance of the derived lineages, as well as the elevated expression of *vim-1* as measured by RT-qPCR (Table 4), was a result of an increase in *vim-1* gene dosage.

### *E. coli* DH5α resistance to ertapenem requires both *vim-1* and mutations of an outer membrane porin

Despite the increased *vim-1* expression in the evolved lineages described above, none of the derived isolates were considered resistant to either ertapenem or meropenem under the tested conditions based on the CLSI guidelines (Table 3). To evolve higher ertapenem resistance in *E. coli* DH5α, an *E. coli* DH5α strain carrying the enlarged pBBR1mcs-5::*vim-1* plasmid isolated from one of the *K. grimontii* KP5022-derived lineages was subjected to ALE and sub-cultured six times in the presence of increasing concentrations of ertapenem, following which individual isolates were collected from two replicate cultures. Antibiotic susceptibility testing confirmed that the derived isolates displayed very high resistance to all three tested antibiotics (ceftazidime, meropenem and ertapenem) (Table 3).

Curing of the *vim-1*-containing plasmid from the derived lineages resulted in close to ancestral levels of resistance, while transfer of the *vim-1*-containing plasmid to otherwise WT *E. coli* DH5α resulted in little resistance to meropenem or ertapenem (Table 3). Together, these results suggest that the high ertapenem resistance of the derived lineages is dependent both on the presence of *vim-1* and at least one additional mutation on the chromosome. To identify the chromosomal mutations responsible for elevated ertapenem resistance, whole-genome sequencing of two derived isolates (strains EcGQ0088 and EcGQ0089; Table 3), as well as the ancestral strain (strain EcGQ0079; Table 3), was performed. Mapping of the EcGQ0088 sequencing reads to the EcGQ0079 reference genome identified a single polymorphism: an in-frame deletion of six nucleotides within *ompC*, which encodes outer membrane porin C, a transmembrane protein that facilitates the transport of beta-lactam antibiotics across the outer membrane [73]. Similarly, EcGQ0089 contained only a single mutation: a 26 kb deletion that spanned *ompC*, among other genes. Overall, these results indicate that high levels of *vim-1*-mediated ertapenem resistance in *E. coli* DH5α are dependent on loss-of-function mutations within *ompC*.

## Discussion

### The gene *vim-1* is disseminated clonally and by plasmid-mediated HGT in Ontario

Plasmids from all clinical isolates identified here can be linked to those associated with hospital outbreaks in Ontario and Europe and appear to form three distinct clades. The first clade, which forms around the *vim-1* plasmids of *E. coli* F5664 and S2568, contains plasmids belonging to the IncA incompatibility group. The spread of plasmids in this clade, both geographically and across species of bacteria, suggests that plasmids of this cluster are globally distributed and have been spread through HGT. The second clade contained the *vim-1*-containing plasmids of *E. hormaechei* H17629, F48994, *K. pneumoniae* T64870 and two additional plasmids belonging to the multilocus IncR-IncN incompatibility group from Toronto [17]. The third clade was formed around the *vim-1-*containing plasmid from *E. hormaechei* H70375 and four additional plasmids from Toronto [17], all belonging to the IncR incompatibility group. For both of these clades, the detection of similar plasmids in isolates from two cities within Ontario, Canada, but not yet elsewhere at the time this analysis was performed, suggests that these plasmids have been spread across species via HGT within Ontario. However, it is unknown whether IncR plasmids are transferable. Their broad host range suggests that they should be transferable [17,74], but this plasmid complex lacks a transfer system and relaxase necessary for mobilization [17,75, 76]. Therefore, it is possible that plasmids in the third clade can be found in *E. hormaechei* isolates across Ontario due to vertical transfer and subsequent evolution, but such conclusions would require further phylogenomic analysis. Overall, these findings support the current consensus on *vim-1* in Ontario that the gene is spread both clonally and by plasmid-mediated HGT [17,77, 78].

### Genomic background influences VIM-1 activity

Among the six clinical isolates, the MICs of ertapenem ranged from 0.25 µg ml^−1^, which is considered sensitive by CLSI guidelines, to 3 µg ml^−1^, which is considered resistant. Ertapenem resistance was even lower (0.006 to 0.012 µg ml^−1^) when *vim-1* was cloned and introduced into three common *Enterobacteriaceae* laboratory strains. This phenotypic variation is consistent with previous studies noting phenotypic variability of MBLs when introduced into diverse bacterial hosts [79]. The differences in resistance phenotype do not fall cleanly around species lines, since there are variations in MICs of ertapenem and meropenem between individual *E. coli* and *E. hormaechei* isolates, although in general, the *E. coli* clinical isolates show higher ertapenem resistance than the other four isolates.

The above observations led us to consider possible mechanistic explanations for the phenotypic variability across our six clinical isolates. Since VIM-1 is integron-encoded and appears in identical positions relative to the integron promoter in all six clinical isolates, differences in promoter proximity and promoter strength are unlikely to explain the differences in resistance phenotypes conferred by *vim-1*; indeed, no obvious relationship was observed between resistance and *vim-1* expression as measured by RT-qPCR. However, mRNA abundance and protein periplasmic concentrations are not necessarily correlated, and it has been noted that variation in MBL periplasmic concentration contributes to the phenotypic variation of MBLs across strains [79].

 Another possibility is that the variations in VIM-1-mediated ertapenem resistance across the strains are due to epistatic interactions between *vim-1* and other genomic loci. Previous studies have observed that high resistance to ertapenem and other carbapenems depends on the presence of a β-lactamase paired with reduced outer membrane permeability [71,80,84]. With one exception [80], those studies focused on variations in membrane permeability in strains with ESBLs rather than strains with true carbapenemases (like VIM-1). Examining the genomes of our six clinical isolates for outer membrane porins revealed that four of the six strains had at least two copies of *ompC* and that the *E. hormaechei* strains also carried *ompD*. In addition, the OmpC amino acid sequences of our six isolates were highly variable, sharing between 62.9 and 100% sequence identity (Fig. S4), and differed in number. Interestingly, the two *E. hormaechei* clinical isolates that were sensitive to carbapenems (isolates H17629 and H70375) each encode a highly similar second OmpC protein; this protein is absent in *E. hormaechei* F48994, which was resistant to carbapenems. Thus, we speculate that variation in membrane permeability, due to differences in the sequences and complement of outer membrane proteins, contributed to the differences in ertapenem resistance observed in our clinical isolates. Although experimental studies are required to confirm this hypothesis, our results nevertheless suggest that reduced membrane permeability is likely an important factor impacting carbapenem resistance even in isolates with true carbapenemases.

### VIM-1 alone was not sufficient to confer carbapenem resistance

To experimentally explore factors influencing VIM-1-mediated ertapenem resistance, *vim-1* was amplified from a *vim-1*^+^ clinical isolate without its native promoter and expressed on a pBBR1mcs-5 plasmid in the laboratory strains *E. coli* DH5α, MG1655 and *K. grimontii* KP5022. For all strains, the presence of *vim-1* alone was insufficient to confer resistance to the carbapenem antibiotics meropenem and ertapenem, but it did confer resistance to the cephalosporin antibiotic ceftazidime. These results confirmed that *vim-1* was expressed, and the encoded enzyme was active and suggested that *vim-1* alone is a strong resistance determinant for cephalosporin antibiotics (like ceftazidime) but not necessarily for carbapenems like ertapenem and meropenem. However, we cannot rule out that the lack of strong resistance against carbapenems might have been due, at least in part, to expression of *vim-1* from a *lac* promoter on the broad-host-range plasmid pBBR1mcs-5, which may not reflect the natural expression levels or regulatory mechanisms of *vim-1*.

 ALE was used to identify mutations increasing ertapenem resistance in the laboratory strains carrying *vim-1*. In all cases, the primary mutation that we identified was an increase in the *vim-1* copy number, and thus expression. Although the increase in *vim-1* expression increased the concentration of ertapenem that the strains could survive in the liquid-based ALE experiments, this mutation had little impact on ertapenem MIC as determined by plate-based assays. We speculate that the difference between the liquid-based and agar-based assays is that in the liquid assays, the concentration of ertapenem could have constantly decreased over time as VIM-1 degraded the antibiotic, potentially allowing growth once the concentration was sufficiently low. On the other hand, we speculate that the concentration of ertapenem may not have changed significantly in the plate-based assay due to the potential for diffusion of the antibiotic throughout the plate.

 On the other hand, strong VIM-1-mediated ertapenem resistance in the plate-based assay was observed when *ompC*, encoding an outer membrane porin, was mutated or deleted, although these mutations came with a trade-off of reduced growth in the absence of antibiotics. This is consistent with previous studies showing synergistic effects of pairing an ESBL with reduced outer membrane permeability [80,84]. In addition, an *E. coli* DH5α ALE-derived isolate containing an *ompC* mutation that was cured of its *vim-1*-containing plasmid retained low, but slightly elevated, resistance to meropenem (~6-fold increase in MIC) and ertapenem (~40-fold increase in MIC) compared with WT *E. coli* DH5α (Table 3). This result is consistent with *ompC* mutation alone slightly increasing carbapenem resistance, and that the high carbapenem resistance of the ALE-derived isolates was due to epistatic interactions of *vim-1* with *ompC* mutations. Porins are pore-forming proteins with a β-barrel structure that allow for the passive transport of hydrophilic compounds across the bacterial outer membrane [85]. OmpC is a non-specific porin that plays a role in both membrane integrity and antibiotic transport [86], meaning its mutation or deletion likely resulted in reduced transport of β-lactam antibiotics across the outer membranes, thereby reducing their concentration in the periplasm to a level that does not overwhelm VIM-1.

## Conclusions

Here, we describe six *Enterobacterales* clinical isolates carrying VIM-1 on horizontally transmissible plasmids. These plasmids appear to be globally distributed and to have spread both clonally and via plasmid-mediated HGT. In addition, the six isolates vary in carbapenem resistance, which we hypothesize is driven primarily by variations in outer membrane permeability. In support of this, we observed that transfer of *vim-1* to common laboratory strains failed to result in ertapenem resistance unless paired with mutation or deletion of *ompC* encoding an outer membrane porin. This result is consistent with studies of strains expressing ESBLs, although the requirement for reduced outer membrane permeability for VIM-1-mediated ertapenem resistance has been less studied. Finally, these results further highlight that PCR-based detection of *vim-1* is not necessarily sufficient to demonstrate that an isolate is resistant to carbapenems without functional verification. While PCR for *vim-1* is a useful screening tool, phenotypic susceptibility testing remains important for guiding carbapenem therapy.

## Supplementary material

10.1099/mic.0.001646 Uncited Supplementary Material 1.

## Abbreviations

ALE: adaptive laboratory evolution
AMPs: antimicrobial peptides
AMR: antimicrobial resistance
ARGs: antimicrobial resistance genes
ESBLs: extended-spectrum β-lactamases
gDNA: genomic DNA
HGT: horizontal gene transfer
LB: lysogeny broth
MBLs: metallo-β-lactamases
MH: Mueller–Hinton
NCBI: National Center for Biotechnology Information
NEB: New England Biolabs
OD_600_: OD at 600 nm
ONT: Oxford Nanopore Technologies
PLSDB: plasmid database
RT-qPCR: reverse transcriptase quantitative PCR
TIBDN: Toronto Invasive Bacterial Diseases Network
VIM: Verona Integron-encoded Metallo-β-lactamase
X-gal: 5-bromo-4-chloro-3-indolyl-β-d-galactopyranoside
