# Supplementary material for: Variations in carbapenem resistance associated with the VIM-1 metallo-β-lactamase across the Enterobacterales
Source: Microbiology (Reading). 2026 Jan 21;172(1):001646. doi: 10.1099/mic.0.001646 (PMC12824645; doi:10.1099/mic.0.001646)
Supplement: Uncited Supplementary Material 1. [file mic-172-01646-s001.pdf]

Supplementary Material for
Variations in carbapenem resistance associated with the VIM-1 metallo-β-lactamase across the order *Enterobacterales*

Mia Rondinelli, Sabhjeet Kaur, Owen A. Ledwell, Henry Wong, Prameet M. Sheth, George C. diCenzo

George C. diCenzo
Email: george.dicenzo@queensu.ca

This PDF file includes

Tables S1 to S3 (pages 2-5)
Figures S1 to S4 (pages 6-9)

Table S1. Oligonucleotides used in this study.

Primer name	Sequence	Application
MR001	5'-CCC CAA GCT TCT AGG TTA TGG AGC AGC AAC GAT GTT AC	Forward primer for amplifying *vim-1* of *E. coli* F5446 (encapsulates *attC* site on 3' end)
MR003	5'-GCG CGG ATC CGC AAA AGT CCC GCT CCA AGC	Reverse primer for amplifying *vim-1* of *E. coli* F5446
SK003	5'-ACT CCT ACG GGA GGC AGC AGT	Forward primer for qPCR of 16S rRNA genes.
SK004	5'-TAT TAC CGC GGC TGC TGG C	Reverse primer for qPCR of 16S rRNA genes.
GD018	5'-ATG GTC TCA TTG TCC GTG ATG	Forward primer for screening VIM in *Enterobacteriaceae* clinical isolates and for qPCR.
GD019	5'-GTC ATG AAA GTG CGT GGA GA	Reverse primer for screening VIM in *Enterobacteriaceae* clinical isolates and for qPCR.

Restriction sites added to the 5' end of primers to facilitate clonings are underlined.

Table S2. Genome assembly and annotation statistics.

Characteristic	*E. coli* F5446	*E. coli* S2568	*E. hormaechei* F48994	*E. hormaechei* H17629	*E. hormaechei* H70375	*K. pneumoniae* T64870
Genome size (bp)	5,382,174	5,388,229	5,160,253	4,988,147	5,240,385	5,768,527
No. of scaffolds	4	5	7	3	6	3
G+C content (%)	50.73%	50.75%	54.77%	55.18%	54.49%	57.02%
No. of protein coding genes	4,878	4,884	4,731	4,450	4,798	5,327
Scaffolds N50	1	1	1	1	1	1
Scaffolds L50	5,028,008	5,044,934	4,722,177	4,794,996	4,680,518	5,452,037
Completeness (%)	99.97	99.97	99.87	99.71	99.51	99.84
Contamination (%)	0.25	0.06	0.22	0.98	0.82	0.33

Table S3. Summary of resistance genes identified in the clinical isolates using the Resistance Gene Identifier (RGI) of the Comprehensive Antibiotic Resistance Database (CARD).

Gene name	AMR gene family	Resistance mechanism	F5446	S2568	F48994	H17629	H70375	T64870
AAC(6')-Ib-cr5	AAC(6') aminoglycoside acetyltransferase	Antibiotic inactivation	✓		✓		✓	
AAC(6')-Ib'	AAC(6') aminoglycoside acetyltransferase	Antibiotic inactivation			✓	✓		
AAC(6')-Ib4	AAC(6') aminoglycoside acetyltransferase	Antibiotic inactivation	✓	✓	✓	✓	✓	✓
ACT-24	ACT beta-lactamase	Antibiotic inactivation			✓			
aadA3	ANT(3'') nucleotidyltransferase	Antibiotic inactivation					✓	
aadA5	ANT(3'') nucleotidyltransferase	Antibiotic inactivation	✓	✓				
aph(3')-XV	APH(3') aminoglycoside O-phosphotransferase	Antibiotic inactivation	✓	✓				
aphA15	APH(3') aminoglycoside phosphorylase	Antibiotic inactivation	✓	✓				
LptD	ATP-binding cassette (ABC) antibiotic efflux pump	Antibiotic efflux						✓
msbA	ATP-binding cassette (ABC) antibiotic efflux pump	Antibiotic efflux	✓	✓				
CTX-M-15	CTX-M beta-lactamase	Antibiotic inactivation	✓	✓	✓		✓	
LAP-2	LAP beta-lactamase	Antibiotic inactivation			✓			
mphA	Macrolide phosphotransferase (MPH)	Antibiotic inactivation	✓	✓	✓			✓
emrB	Major facilitator superfamily (MFS) antibiotic efflux pump	Antibiotic efflux	✓	✓				
emrR	Major facilitator superfamily (MFS) antibiotic efflux pump	Antibiotic efflux	✓	✓				
emrY	Major facilitator superfamily (MFS) antibiotic efflux pump	Antibiotic efflux	✓	✓				
K. pneumoniae KpnE	Major facilitator superfamily (MFS) antibiotic efflux pump	Antibiotic efflux						✓
K. pneumoniae KpnF	Major facilitator superfamily (MFS) antibiotic efflux pump	Antibiotic efflux						✓
mdtG	Major facilitator superfamily (MFS) antibiotic efflux pump	Antibiotic efflux	✓	✓				
mdtH	Major facilitator superfamily (MFS) antibiotic efflux pump	Antibiotic efflux	✓	✓				
qacEdelta1	Major facilitator superfamily (MFS) antibiotic efflux pump	Antibiotic efflux	✓	✓	✓	✓	✓	✓

Continued on next page

Gene name	AMR gene family	Resistance mechanism	F5446	S2568	F48994	H17629	H70375	T64870
evgA	Major facilitator superfamily (MFS) antibiotic efflux pump, resistance-nodulation-cell division (RND) antibiotic efflux pump	Antibiotic efflux	✓	✓				
H-NS	Major facilitator superfamily (MFS) antibiotic efflux pump, resistance-nodulation-cell division (RND) antibiotic efflux pump	Antibiotic efflux	✓	✓				
OXA-1	OXA beta-lactamase	Antibiotic inactivation	✓		✓		✓	
OXA-9	OXA beta-lactamase	Antibiotic target replacement			✓			
QnrB1	Quinolone resistance protein (qnr)	Antibiotic target protection					✓	
QnrS1	Quinolone resistance protein (qnr)	Antibiotic target protection			✓	✓	✓	✓
baeR	Resistance-nodulation-cell division (RND) antibiotic efflux pump	Antibiotic efflux	✓	✓				
oqxA	Resistance-nodulation-cell division (RND) antibiotic efflux pump	Antibiotic efflux						✓
marA	Resistance-nodulation-cell division (RND) antibiotic efflux pump, General Bacterial Porin with reduced permeability to beta-lactams	Antibiotic efflux, reduced permeability to antibiotic	✓	✓				
SHV-12	SHV beta-lactamase	Antibiotic inactivation	✓	✓				
SHV-28	SHV beta-lactamase	Antibiotic inactivation						✓
sul1	Sulfonamide resistant sul	Antibiotic target replacement	✓	✓	✓	✓	✓	✓
sul2	Sulfonamide resistant sul	Antibiotic target replacement					✓	
TEM-1	TEM beta-lactamase	Antibiotic inactivation			✓		✓	
dfrA14	Trimethoprim resistant dihydrofolate reductase dfr	Antibiotic target replacement	✓	✓	✓	✓	✓	✓
dfrA15	Trimethoprim resistant dihydrofolate reductase dfr	Antibiotic target replacement			✓			
dfrA17	Trimethoprim resistant dihydrofolate reductase dfr	Antibiotic target replacement	✓	✓				
VIM-1	VIM beta-lactamase	Antibiotic inactivation	✓	✓	✓	✓	✓	✓

F5446 – *E. coli*; S2568 – *E. coli*; F48994 – *Enterobacter hormaechei*; H17629 – *E. hormaechei*; H70375 – *E. hormaechei*; T64870 – *Klebsiella pneumoniae*.

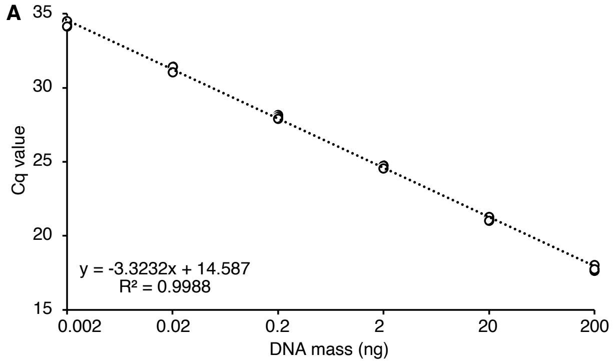

Figure S1. qPCR standard curves. Standard curves are shown for the primers (**A**) GD018 and GD019 targeting *vim-1*, and (**B**) SK003 and SK004 targeting the 16S rRNA gene. Technical triplicates were performed for each tested cDNA concentration and are plotted separately. The Y-axis represents the total mass of cDNA added to each reaction. The dashed line shows the linear line-of-best-fit. Based on the slope, the amplification efficiency for the *vim-1* primers is 99.9% while the amplification efficiency for the 16S rRNA gene primers is 94.1%.

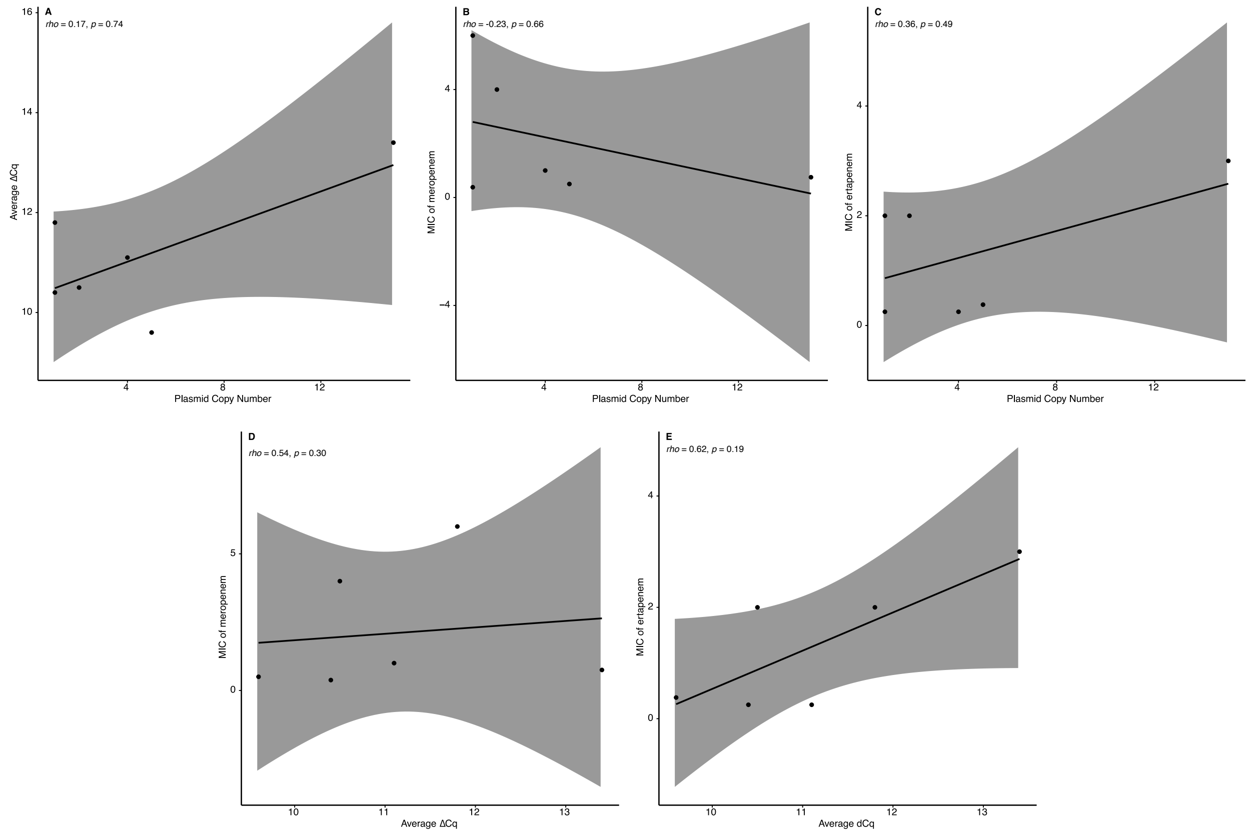

Figure S2. Correlation between the *vim-1*-containing plasmids or *vim-1* expression and resistance to carbapenems in the six *Enterobacterales* clinical isolates. Scatterplots with Spearman correlations are shown for: (**A**) copy number of the *vim-1*-containing plasmids and *vim-1* expression measured as average ΔCq from three biological replicates (higher ΔCq values indicate lower expression); (**B**) copy number of the *vim-1*-containing plasmids and the minimum inhibitory concentration (MIC) of meropenem; (**C**) copy number of the *vim-1*-containing plasmids and the MIC of ertapenem; (**D**) *vim-1* expression measured as average ΔCq from three biological replicates (higher ΔCq values indicate lower expression) and the MIC of meropenem; and (**E**) *vim-1* expression measured as average ΔCq from three biological replicates (higher ΔCq values indicate lower expression) and the MIC of ertapenem.

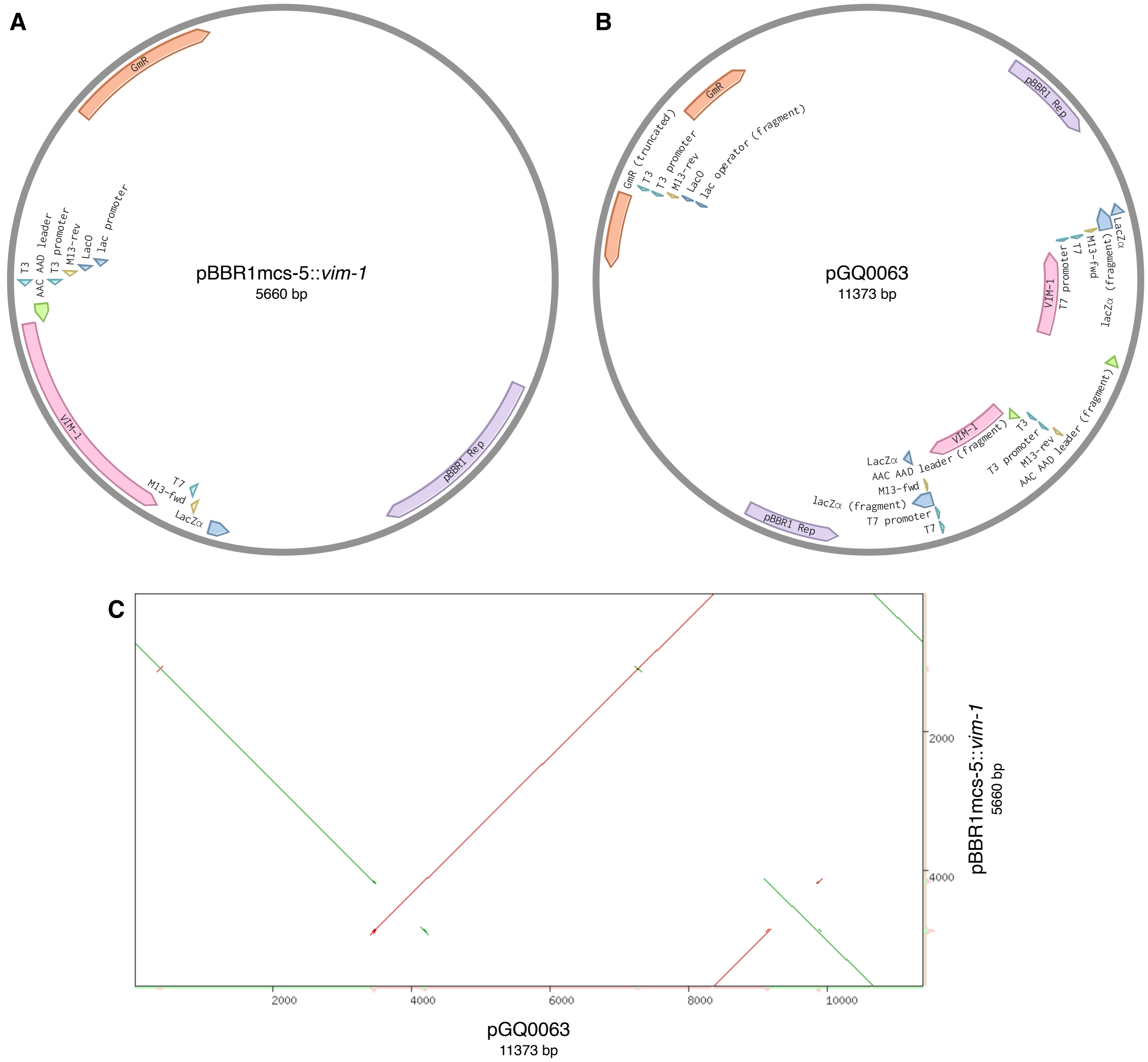

Figure S3. Adaptive laboratory evolution lead to duplication of the *vim-1*-containing pBBR1mcs-5 plasmids. Plasmid maps are shown for (**A**) pBBR1mcs-5 expressing *vim-1*, and (**B**) the pBBR1mcs-5::*vim-1* plasmid isolated from a *K. grimontii* derived isolate following adaptive laboratory evolution. The plasmid isolated from the derived isolate is approximately double the size of the original plasmid and carries two copies of *vim-1*. (**C**) A dot plot is shown, comparing the sequence of pBBR1mcs-5::*vim-1* (Y-axis) to the plasmid isolated from the derived isolate (X-axis), showing that the plasmid from the derived isolate appears to have formed as a result of homologous recombination between two copies of pBBR1mcs-5::*vim-1*.

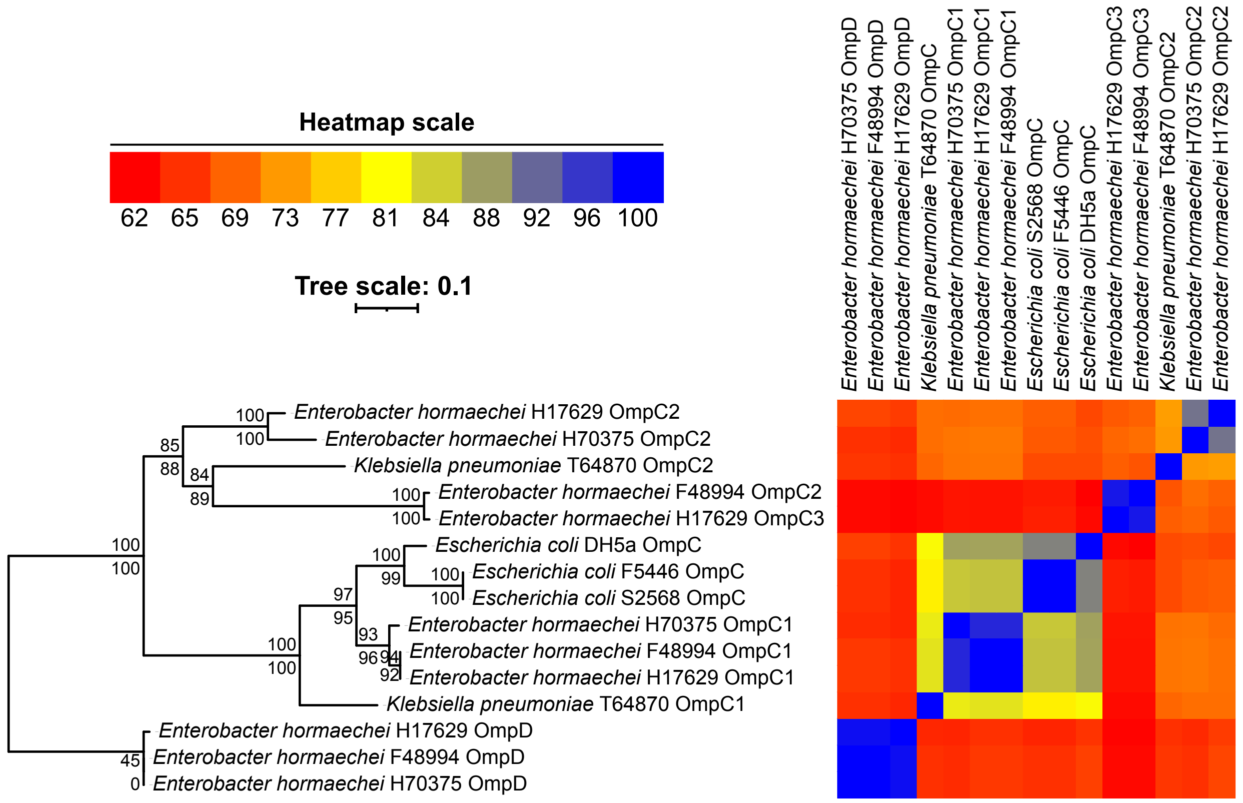

Figure S4. Phylogeny of OmpC and OmpD of several strains from the order *Enterobacterales*. OmpC and OmpD proteins were extracted from the proteomes of *Escherchia coli* DH5α and the six clinical isolates described in this study, based on their annotated gene names. On the left is an unrooted maximum likelihood phylogeny showing the evolutionary relationships between the proteins. The numbers on the nodes indicate the ultra-fast bootstrap values (top numbers) and the SH-aLRT support values (bottom numbers), both calculated from 1000 replicates. The scale bar represents the average number of amino acid substitutions per site. On the right is a heatmap showing the percent identity between each pair of proteins, as calculated by Clustal Omega, with colour representing percent identity as shown by the heatmap scale.
